# Evidence for current recommendations concerning the management of foot health for people with chronic long-term conditions: a systematic review

**DOI:** 10.1186/s13047-017-0232-3

**Published:** 2017-11-22

**Authors:** Katherine Edwards, Alan Borthwick, Louise McCulloch, Anthony Redmond, Rafael Pinedo-Villanueva, Daniel Prieto-Alhambra, Andrew Judge, Nigel Arden, Catherine Bowen

**Affiliations:** 1 Nuffield Department of Orthopaedics, Rheumatology and Musculoskeletal Sciences, University of Oxford, Oxford, UK; 2 Faculty of Health Sciences, University of Southampton, Highfield Campus Building 45, University Road, Southampton, Hampshire SO17 1BJ UK; 3 Faculty of Medicine and Health, Leeds Institute of Rheumatic and Musculoskeletal Medicine, University of Leeds, Leeds, UK

**Keywords:** Foot, Feet, Footcare, Foot health, Podiatry, Chronic conditions, Guidelines

## Abstract

**Background:**

Research focusing on management of foot health has become more evident over the past decade, especially related to chronic conditions such as diabetes. The level of methodological rigour across this body of work however is varied and outputs do not appear to have been developed or translated into clinical practice. The aim of this systematic review was to assess the latest guidelines, standards of care and current recommendations relative to people with chronic conditions to ascertain the level of supporting evidence concerning the management of foot health.

**Methods:**

A systematic search of electronic databases (Medline, Embase, Cinahl, Web of Science, SCOPUS and The Cochrane Library) for literature on recommendations for foot health management for people with chronic conditions was performed between 2000 and 2016 using predefined criteria. Data from the included publications was synthesised via template analysis, employing a thematic organisation and structure. The methodological quality of all included publications was appraised using the Appraisal for Research and Evaluation (AGREE II) instrument. A more in-depth analysis was carried out that specifically considered the levels of evidence that underpinned the strength of their recommendations concerning management of foot health.

**Results:**

The data collected revealed 166 publications in which the majority (102) were guidelines, standards of care or recommendations related to the treatment and management of diabetes. We noted a trend towards a systematic year on year increase in guidelines standards of care or recommendations related to the treatment and management of long term conditions other than diabetes over the past decade. The most common recommendation is for preventive care or assessments (e.g. vascular tests), followed by clinical interventions such as foot orthoses, foot ulcer care and foot health education. Methodological quality was spread across the range of AGREE II scores with 62 publications falling into the category of high quality (scores 6–7). The number of publications providing a recommendation in the context of a narrative but without an indication of the strength or quality of the underlying evidence was high (79 out of 166).

**Conclusions:**

It is clear that evidence needs to be accelerated and in place to support the future of the Podiatry workforce. Whilst high level evidence for podiatry is currently low in quantity, the methodological quality is growing. Where levels of evidence have been given in in high quality guidelines, standards of care or recommendations, they also tend to be strong-moderate quality such that further strategically prioritised research, if performed, is likely to have an important impact in the field.

**Electronic supplementary material:**

The online version of this article (10.1186/s13047-017-0232-3) contains supplementary material, which is available to authorized users.

## Background

Chronic conditions represent a global health burden [1]. Their impact on the societal and individual level has led to the primary prevention of chronic conditions being designated a priority area for UK National Health [2, 3]. Current strategies include exercise as a core component, but assume that individuals will not be impeded by conditions involving pain and disability [4, 5]. An unmet need for the management of foot and ankle problems is acknowledged in the current literature [6–12]. To reflect the scale of the problem, around 8% of a General Practitioner’s (GP’s) caseload is reportedly related to foot and ankle problems despite only a relatively small proportion of the population seeking treatment [13, 14]. It is further reported that one in three people over 65 years are unable to manage basic personal foot care such as cutting their toenails [15].

Despite this need for the management of foot problems, provision of podiatry services is an area that lacks guidance. Feedback from people who have chronic conditions suggests that they are not accessing care for foot problems [12, 16, 17] and that they are confused over referral pathways to podiatry services [9, 16, 18]. There appears to be a general lack of understanding of what podiatry services can do on the part of both patients and non-podiatric clinicians [12, 16].

There has been increased attention to evidence informed medicine and the desire for reproducibility in choice of foot-care tailored to individual patient needs [6, 9, 13, 18–22]. Policy decision makers use available evidence to make their decisions and to be informed, while patients need up-to-date information about treatment options so that they are able to weigh the benefits against the risks of their treatment options [23]. Guidelines, standards of care and recommendations for practice have the intention of improving the process and outcomes of healthcare and to optimise resource utilisation [24]. They are usually based on the synthesis of the best, most recent evidence that is distilled into a convenient, usable format that can help clinicians integrate evidence into their practice as well as direct policy makers to make their decisions on foot health services [25].

Guidelines, standards of care and recommendations for management of foot health have become more evident over the past decade, particularly in the management of foot problems related to the manifestations of diabetes [22]. The impact on local and national decision making of these, however, appears to be negligible. This is disappointing and it may be due to methods used and the quality of the evidence utilised in the development of guidelines related to foot health varying greatly [21, 22]. It is possible that those based on incomplete or biased evaluation of the literature can lead to inappropriate recommendations, ineffective or harmful practices. It is thus important that the method of development of guidelines, standards of care and clinical practice recommendations for foot care for people who have chronic conditions is rigorous and open to scrutiny. To our knowledge, no other data exists that has scrutinised the evidence for, or against interventions to manage foot health in patients with long-term chronic conditions.

The aim of this investigation was to systematically review the literature related to guidelines, standards of care and current recommendations concerning the management of foot health for people with chronic conditions. The findings of this study will be of interest to podiatrists and health care professionals by identifying the evidence-based behind interventions for foot health management, which can then be used to support and guide practice. Specific objectives were:

1. To provide a narrative synthesis on the specific characteristics of guidelines and recommendations for foot health management using criteria from the Preferred Reporting Items for Systematic Reviews and Meta-Analyses (PRISMA) checklist [26].
2. To assess the methodological quality of included studies against the Appraisal of Guidelines for Research and Evaluation (AGREE II) document [27].
3. To determine the level of evidence used in guidelines and recommendations for foot health management.

## Method

### Search strategy

Search criteria for the systematic review were identified using the “PICO” statement. Search terms were defined by three experts in podiatry (CB, AR, AB) and the final list agreed through a consensus meeting. Electronic databases (Medline, Embase, Cinahl, Web of Science, SCOPUS and The Cochrane Library) were searched for English language publications and translations reporting recommendations for the management of foot health for people with chronic conditions between 2000 and 2016. The year 2000 was considered an appropriate point to commence the literature search, given the establishment of degree education in podiatry from 1993, and the subsequent development of postgraduate and post-doctoral qualifications and research skills within the profession.

Searches were performed using the following text word terms and MeSH headings: foot, chronic, chronic disease, long term care, neurodegenerative diseases, multiple sclerosis, arthritis, lung diseases, emphysema, diabetes mellitus, hypertension, cerebrovascular disorders, myocardial ischemia, heart failure, colonic diseases, human immunodeficiency virus, osteoporosis, fibromyalgia, neoplasms, mental disorders, depression, asthma, chronic obstructive pulmonary disease, thyroid diseases, hyperlipidaemia, psoriatic arthritis, gout, ankylosing spondylitis, colitis, scleroderma, practice guidelines, algorithms.

Hand searches of bibliographic references identified additional publications. Grey literature was included based on an initial search using the terms foot, feet and podiatry and rerun in conjunction with the terms guideline, recommendation and management to ensure that the search had captured all relevant sources. Publications identified through the grey literature and hand searches were not restricted by date. The systematic literature search was facilitated by a medical librarian at the Bodleian library, University of Oxford. The full search strategy can be seen in Additional file 1: Appendix A.

### Selection criteria

A publication was included if it met all the following criteria:

## Includes adults with chronic conditions

(Chronic conditions are here defined as long-term conditions, e.g. diabetes, stroke, gout, rheumatoid arthritis and osteoarthritis).

1. Includes the management of foot health
2. Provides recommendations for the management of foot health, e.g. guidelines, guidance documents, standards of care, treatment algorithms and clinical pathways.

Excluded publications were those that did not fulfil the above criteria on population group, were not related to the management of foot health, such as technical and methodological papers, and were not formal publications presenting guidelines or recommendations (audit reports, conference abstracts, opinion letters, critical review summaries, press and media releases, and briefing documents). Literature identified from the electronic search strategy was excluded if published outside the date range 2000 to 2015.

Two independent reviewers (KE and LM) selected eligible publications based on titles and abstracts. Potentially relevant publications were subject to full-text screening. Disagreement between reviewers was resolved by consultation with a third reviewer from a panel of qualified podiatrists and experts in the field of podiatry and foot and ankle research (CB, AB, and AR). Data was extracted from the identified literature based on the criteria given in the PRISMA (2009) checklist [26]. The PRISMA statement consists of a set of evidence-based criteria which reflect the minimum standard of reporting in systematic reviews and meta-analyses [26].

### Quality assessment

Three independent expert reviewers (CB, AB & KE) assessed all the included publications (July 2016) for quality using the AGREE II checklist [27]. The AGREE II checklist was developed to address issues of variability in the quality of practice guidelines and is used internationally as a means of assessing the standard of methodology used in the approach to guideline development. The checklist evaluates the quality of 23-items across six domains that relate to aspects of the guideline development through assessment of six domains including, scope and purpose, involvement of stakeholders, developmental rigour, presentation, applicability and editorial independence. An overall score of 7 reflects that the guideline strongly agrees with most of the AGREE II criteria, while an overall score of 1 shows a weak agreement. Whilst the consortium for the development of the AGREE II criteria set the domain scores, they did not set minimum domain scores for high or low quality guidelines. Instead they advise that the decision should be made by the user and guided by the context in which AGREE II is being used. Therefore, through consensus of all authors, we set threshold scores at high quality (scores 6–7), mid-level quality and partially meeting the AGREE II criteria (scores 4–5), low methodological quality (scores 1–3). The scores for all included papers can be seen in Additional file 2: Appendix B.

### Grading of recommendations

Data from the included publications was synthesised via template analysis, employing a thematic organisation and structure. Template analysis is a process by which textual data is organised and analysed according to themes, allowing unstructured textual material to be categorised into meaningful datasets. Indexing sections of text relevant to a theme also permits the development of codes which emerge from the data [28]. Predetermined themes related to foot care and / or podiatry services were established from synthesis of current literature by experts in the field of podiatry (CB, AB) [29, 30] (Table 1). Each theme was assigned a code which when noted within a publication was readily entered into the data extraction sheet. A theme could be present singularly or all codes could exist within the same publication.

**Table 1:** Themes and associated codes used to categorise management of foot health for people with chronic conditions

Code	Theme
A	Core podiatry (nail, corn and callus care)
B	Foot ulcer care
C	Foot health education
D	Preventive care or assessments (vascular, neurological, musculoskeletal, dermatological)
E	Preventive care advancements or assessments (diagnostic ultrasound, ABPIs, advanced training or skills)
F	Clinical interventions (provision of foot orthoses, insoles, nail surgery, injection therapies, physical therapies)
G	Podiatric surgery
H	Other
I	Pain medication
J	Antibiotics
K	Orthopaedic surgery
L	Antifungals
M	Footwear assessment

Where stated, the level of evidence supporting the recommendations related to foot health management was extracted and interpreted according to the GRADE (Grading of Recommendations Assessment, Development and Evaluation) system [31]. The GRADE system classifies the quality of evidence as high, moderate, low, and very low (Table 2). For example, evidence based on randomised controlled trials is ranked as high quality evidence and expert opinion, case series is ranked as the lowest level of evidence [31]. The reasoning for this more rigorous assessment was that, in some cases, while the overall publication quality may have been low or average based on the AGREE II criteria [27], some evidence underpinning the publication may in fact be of high quality or vice-versa.

**Table 2:** Grading of Recommendations Assessment, Development and Evaluation system. Adapted from Guyatt et al. 2008 [31]

Grading	Publication type	Recommendations
*High quality*	Meta-analyses, systematic reviews of RCTs, or RCTs with a low risk of bias	Further research is very unlikely to change our confidence in the estimate of effect
*Moderate quality*	High quality systematic reviews of case control or cohort studies; High quality case control or cohort studies with a very low risk of confounding or bias and a high probability that the relationship is causal; Well-conducted case control or cohort studies with a low risk of confounding or bias and a moderate probability that the relationship is causal	Further research is likely to have an important impact on our confidence in the estimate of effect and may change the estimate
*Low quality*	Case control or cohort studies with a high risk of confounding or bias and a significant risk that the relationship is not causal	Further research is very likely to have an important impact on our confidence in the estimate of effect and is likely to change the estimate
*Very low quality*	Non-analytic studies, e.g. case reports, case series; Expert opinion	Any estimate of effect is very uncertain

## Results

The search strategy retrieved 5379 citations from electronic and grey literature sources. In total, 1938 duplicate citations were removed between the electronic and grey literature sources. Results from the electronic search were then subject to title screening according to the review selection criteria with 856 citations being excluded; results from the grey literature were not subject to title screening as this was deemed inappropriate due to the use of short titles for the majority of articles, and all were put forward for screening by abstract. Following abstract screening, 2368 citations were found ineligible with 122 citations being included from hand searching the bibliographies of excluded papers. The search process was then repeated for 2015 to 2016 to ensure that the findings of the review would be as up-to-date as possible, and this resulted in the identification of an additional 14 publications. From full-text screening, 187 articles were considered ineligible, and the remaining 166 publications were included in the final review.

The abstracts of all publications found were exported from the databases and collated into a Microsoft® Excel (2010) spreadsheet (Microsoft Corporation, Redmond, Washington, USA) for scoring for inclusion (1) or exclusion (0).

Screening by abstract resulted in the exclusion of 2358 citations. An additional 122 relevant citations were also identified from hand searching bibliographies of any publications excluded at this stage. Of the 339 citations that were put forward for full-text screening, 152 were included in the final review.

**Fig. 1:**
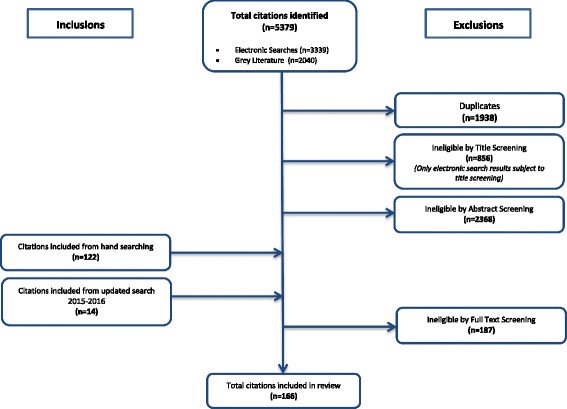
PRISMA flow diagram of the literature search

The full text version of all publications found were exported from the databases and collated into a second Microsoft® Excel (2010) spreadsheet for scoring for inclusion (1) or exclusion (0). Four reviewers (CB, AB, LM & KE) independently screened the full text citations against the inclusion criteria. All publications that met the inclusion criteria were selected, however only the most recent versions were included such that 152 were put forward for review. The literature search process was repeated for any publications that had been released until September 2016 to make the review as up-to-date as possible. An additional 14 publications were identified, bringing the final total up to 166. Fig. 1 illustrates the flow of the literature selection process.

### Descriptive characteristics

Of the 166 included publications, 74 (45%) were from the grey literature, 65 (39%) were from electronic databases and 27 (16%) were from hand searches.

#### Publication date

**Fig. 2:**
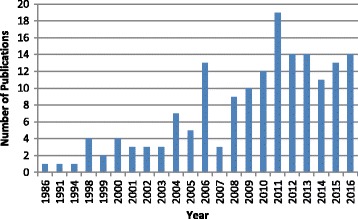
Distribution of Included Articles by Year of Publication

The 166 included papers were published between 1986 and 2016; of these, the majority (11.5%) were published in 2011. Prior to 2000, the studies represented are those identified from the grey literature for which no date criteria were imposed. Importantly, the distribution of guidelines, standards of care and recommendations concerning the management of foot health in people with chronic conditions shows a steadily increasing trend over time (Fig. 2).

#### Location

**Fig. 3:**
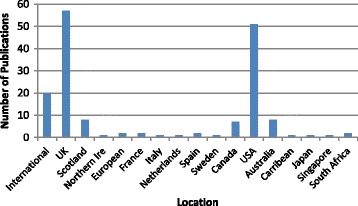
Geographical Distribution of Included Studies

Most (39.8%) of the 166 included publications were based in, or included, the United Kingdom (Fig. 3). Another 51 (30.7%) involved the USA, of which one was in collaboration with Europe. Twenty (12.1%) were international publications and involved the collaboration of authors or institutions from two or more countries, of which at least one was based outside of Europe. Nine (5.4%) were based in Europe, with those remaining being from various other non-European countries including, Australia (4.8%), Canada (4%), South Africa (1.2%), the Caribbean (0.6%), Japan (0.6%) and Singapore (0.6%). The review inclusion criteria selected only those guidelines, recommendations and standards of care that were in English or for which and English translation was available, therefore numbers of available publications from outside the UK and USA concerning foot health may well be higher than those shown.

#### Publication type

**Fig. 4:**
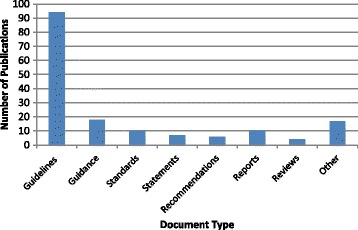
Distribution of Included Studies by Publication Type

The majority (56.6%) of the 166 included publications were reported as guidelines; this included Global or National guidelines, clinical guidelines, best practice guidelines, evidence-based guidelines and guideline summaries (Fig. 4). Among the remaining papers; 18 (10.8%) related to guidance documents or summaries; ten (6.0%) were reports; another ten (6.2%) were in relation to standards (e.g. quality standards, standards of care); seven (4.2%) were statements of position, best practice or consensus; six (3.6%) provided recommendations and four (2.4%) were reviews. Seventeen (10.2%) were classified as ‘other’, which consisted of individual papers that included but were not limited to the following types; case studies, action plans, model of practice, prevention tools, treatment algorithms and cost-effectiveness analyses.

#### Disease type or condition

**Fig. 5:**
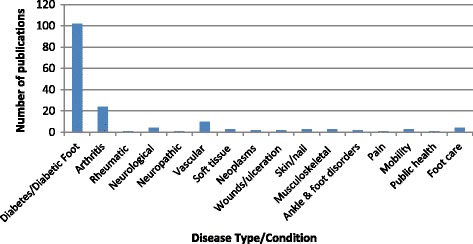
Distribution of Included Studies according to Disease Type or Condition

Of the 166 included publications, the majority (61.5%) were guidelines, standards of care or recommendations related to the treatment and management of diabetes or the diabetic foot (Fig. 5). Of the remaining articles, 24 (14.5%) were concerned with types of arthritis, including rheumatoid arthritis (8.4%), osteoarthritis (1.8%), gout (1.8%), psoriatic arthritis (1.2%), and other inflammatory arthritis (1.2%). Another ten (6.0%) dealt with peripheral arterial disease (PAD); four (2.4%) with general foot care and neurological conditions, three (1.8%) in relation to musculoskeletal, skin and nail or soft tissue conditions and mobility. Two (1.2%) publications each related to foot and ankle conditions, neoplasms, wounds and ulceration, with only individual papers (0.6%) on rheumatic or neuropathic disease, foot pain or general public health.

### AGREE II quality assessments

**Fig. 6:**
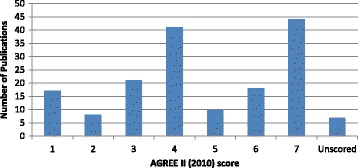
Methodological Scoring of Included Papers against the AGREE II Criteria

Quality assessment of the 166 included publications was carried out using the AGREE II checklist (Fig. 6). We found that the methodological quality was spread across the range of AGREE II scores. Sixty-two publications fell into the category of high quality (scores 6–7); of these the majority were related to diabetes and the diabetic foot (15.7%), and published in the USA (4.8%). Comparatively, 51 publications were considered to be of mid-level quality and partially met the AGREE II criteria (scores 4–5). Forty-six publications were considered to be of low methodological quality (scores 1–3); of these, 31 (18.7%) came from the electronic literature, 14 (8.4%) from the hand searched literature and one (0.6%) from the grey literature.

### Grading of recommendations

**Fig. 7:**
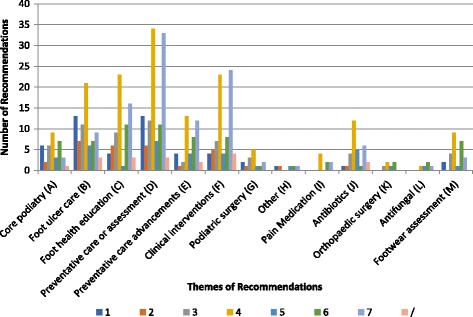
The number of Publications providing recommendations on foot health management and classified according to AGREE II score. Legend: Note: Publications may provide recommendations on multiple topic areas

Synthesis of the themes related to foot health management show that recommendations are made for all themes, but not in all included publications (Fig. 7). The most common recommendation is for preventive care or assessments (e.g. ABI tests), followed by clinical interventions such as foot orthoses, foot ulcer care and foot health education. Comparatively, there is a noticeably lower number of publications containing recommendations for core podiatry and podiatric surgery. Areas with the lowest numbers of recommendations involved themes such as antifungal and pain medication.

Of the included papers that have a quality threshold score of 4 and above for reasonable to high methodological quality (*n* = 113), preventative care and assessments along with clinical interventions and foot health education are themes that are covered the most; on the other hand, comparatively little attention is given here to areas such as core podiatry and podiatric surgery. Recommendations for core podiatry tend to feature the most in publications with an AGREE II grade of 3 or below, indicative of low methodological quality, as do those for podiatric surgery, although these are comparatively few in number overall.

#### Strength of evidence for foot health management

**Fig. 8:**
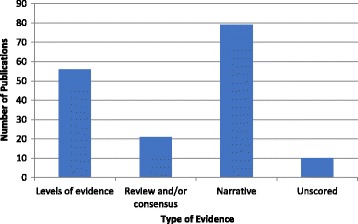
Shows the types of evidence reported by each of the included publications (*n* = 166)

**Fig. 9:**
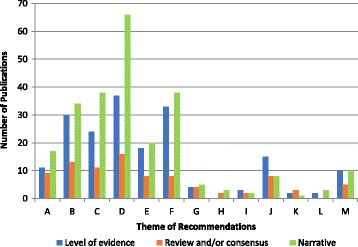
Shows the number of publications and the type of evidence reported according to the theme of the recommendations provided. Legend: Note: Shows the number of publications with at least one recommendation for that particular theme. Publications may give recommendations on multiple themes

The 166 included publications were each screened and the type of evidence they provide is summarised in Fig. 8. This data was then stratified based on the theme of the recommendations given and the AGREE II score of the paper for methodological quality (Additional file 3: Appendix C, Fig. 9). The number of publications providing a recommendation in the context of a narrative but without an indication of the strength or quality of the underlying evidence was high (79 out of 166). The number of publications which indicate that the recommendations provided are based on review and/or consensus was 21 out of 166. Only 56 (33.7%) of the included publications provided levels of evidence to accompany the recommendations given, while ten remained unscored as they provided no recommendations in the context of the themes evaluated.

For the majority of themes, most publications offering a recommendation in the context of that particular theme tend to be more narrative-based; the exception to this includes themes such as pain medication, antibiotics, orthopaedic surgery and footwear assessments for which the numbers of publications providing a level of evidence alongside the given recommendations is slightly higher than those which are written as a narrative.

For most cases, the differences in number between the publications providing evidence-based recommendations to those providing a narrative synthesis is in the region of 1–8 papers; however for themes such as foot health education and clinical interventions (i.e. foot orthoses), the difference between evidence and narrative based publications is more apparent (14+ papers).

From those papers that reported levels of evidence alongside recommendations, this data was then extracted and used to populate a data extraction form (Additional file 4: Appendix D). The levels of evidence were then summarised for each theme according to the AGREE II score of the publication in which they were provided (Additional file 5: Appendix E). Each of the grades and levels of evidence were then interpreted according to the Grading of recommendations assessment, development and evaluation (GRADE) system. Table 3 summarises the breadth of the quality of evidence (i.e. high, moderate, low, very low) behind recommendations concerning foot health management both according to theme and AGREE II score of the initial publication.

**Table 3:** The breadth of the quality of evidence concerning foot health management categorised by theme of recommendations and AGREE II score of publication

Theme	1*	2*	3*	4*	5*	6*	7*	/*
Core podiatry [A]			Low	High, very low	Moderate	Moderate-very low	Low-very low	
Foot ulcer care [B]			High-low	High-very low	High-very low	High-very low	High-very low	High-very low
Foot health education [C]			Low	High-very low		High-very low	High-very low	
Preventative care or assessments [D]			Low	High-very low	High-very low	High-very low	High-very low	
Preventative care or advancements [E]				High-very low	Moderate-low	High-very low	Moderate-very low	
Clinical interventions [F]	Moderate-very low		Moderate-low	High-very low	High-low	High-very low	High-very low	High
Podiatric surgery [G]			Low	Moderate-very low	High-very low			
Other [H]								
Pain medication [I]				Moderate		High, low-very low	Very low	
Antibiotics [J]				Moderate-very low	High-very low	Moderate-low	Very low	Moderate-low
Orthopaedic surgery [K]					High-low	Very low		
Antifungal medication [L]					Moderate	Low		
Footwear assessments [M]				High-moderate		High-very low	High-very low	

* Grading of Recommendations Assessment, Development and Evaluation system

## Discussion

Our systematic search for current evidence for foot health management related to chronic conditions revealed 166 publications. The majority (102) were guidelines, standards of care or recommendations that were focussed on the treatment and management of diabetes. Methodological quality of the included publications ranged from high to low according to the AGREE II rating system [27] suggesting that methods used and the quality of the evidence utilised in the development of such publications vary greatly. Where evidence for foot health management is reported, it is mostly at the level of expert opinion or good clinical practice, although some publications reported supporting evidence that was at a moderate to strong level. Notably, we identified a systematic year on year increase in publications containing supporting evidence for foot health management related to other chronic conditions over the past decade. Additionally, we have built an easily searchable database where the guidelines and recommendations that we identified are categorised and listed so that specific evidence for foot health management and podiatry (as of 2016) can be readily ascertained.

### Demographics

Examining patterns across the 166 publications would suggest that the majority of supporting evidence for foot health management is included within UK guidelines, recommendations or standards of care, followed by the US, Europe and Australia. This is not surprising as our search was limited to English only publications, however of note, within these countries podiatry is a recognised allied health discipline [32, 33]. Podiatry within the UK and Australia has been building a research capacity over the past decade [34] and it is possible that the trend in year on year increases in publications containing supporting evidence for foot health management related to other chronic conditions is linked to this.

### Quality of guidelines

Guidelines, standards of care and recommendations play an important role in synthesising evidence for health care and health policy information however according to the AGREE II consortium [27] the potential benefits are only as good as the quality of the guidelines themselves. We found 62 publications that fell into the category of high quality, 51 were considered to be of mid-level quality and 46 were considered low methodological quality; 7 publications were not given an AGREE II score as the content did not address a sufficient number of the domains being assessed. We found that the methods for guideline development markedly differed according to the stakeholders involved. The high-quality guidelines were largely from government agencies and national professional bodies such as the National Institute for Health and Care Excellence (NICE), the Scottish Intercollegiate Guidelines Network (SIGN), and the National Institutes of Health (NIH), whereas the low-quality guidelines tended to be developed by smaller local or regional health providers and often were single authored publications. This is consistent with other studies that have investigated guidelines specifically for management of foot and ankle problems in rheumatoid arthritis [21] and guidelines for foot screening in diabetes [22].

From our review, we also established that it was rare that a podiatrist or foot care specialist was included as a stakeholder in the higher scoring publications. This is of concern as the recommendations contained within guidelines are defined by the scope of the stakeholders. Stakeholders advise guideline developers in searching for, selecting, critiquing and combining data [24]. Without engagement with podiatrists or foot care specialists it is possible that evidence included in these publications may have incomplete, ill-informed or biased evaluations which in turn can lead to inappropriate recommendations [24]. If podiatrists and foot care specialists are to become involved either as leaders of guideline development or as members of the stakeholder group it is imperative that the methodological approach follows the robust principles, such as those outlined by the AGREE II consortium [27]. The validity of the guideline recommendations should be open to being judged in a rigorous and reproducible way.

### Evidence scores

The strength of recommendations within guidelines, recommendations or standards of care is informed by the quality of the research evidence on which they are based [24, 25]. Following an assessment of the overall quality of the included publications, a more in-depth analysis was carried out that specifically considered the levels of evidence that underpinned the strength of their recommendations concerning foot care, foot health management and/or podiatry. Disappointingly, we found a high number of publications providing a recommendation in the context of a narrative but without an indication of the strength or quality of the underlying evidence for foot care / podiatry. We also found a high number of publications that indicated that the recommendations provided are based on expert opinion or best practice.

Where a level of evidence was given, this tended to be in the higher scoring publications for methodological quality and related to GRADE (Grading of Recommendations Assessment, Development and Evaluation) system [31] as strong-moderate quality. Therefore, some evidence for foot care, foot health management and /or podiatry exists that is underpinned by a limited number of systematic reviews and randomised controlled trials such that those recommendations could apply to most patients in most circumstances [31]. Evidence for preventive care or foot assessments (vascular, neurological, musculoskeletal, dermatological) features in the most publications, followed by foot health education. There is moderate evidence for preventive care advancements or foot assessments (diagnostic ultrasound, ABPIs, advanced training or skills) and clinical interventions (provision of foot orthoses, insoles, nail surgery, injection therapies, physical therapies). Evidence for core podiatry (nail, corn and callus care) is rare and no publications included evidence for podiatric surgery.

According to GRADE, for those themes with emerging good quality evidence, further research, if performed, is likely to have an important impact on confidence in the estimate of the effect and may change the estimate [31]. As the body of work continues to grow, it is thus likely to have an important impact on the evidence for ‘podiatry’ however it is clear that considerable work is still required for key areas that will do well to have a targeted and strategic approach.

### Limitations

The findings of this systematic review need to be considered in the context of several limitations. First the scrutiny for evidence for ‘podiatry’ was determined a priori by the authors as being most accessible from guidelines as they usually incorporate research evidence into their clinical recommendations. In this instance it would have been inappropriate to select publications by single interventions due to the wide range of interventions related to management of foot health / podiatry in long term conditions and the multi-professional nature involved in the interventions. As only English-language publications were included in the review this may have resulted in additional relevant, high-quality evidence from non-English publications being omitted.

The hand searching process also procured a number of publications not identified from the electronic database search. This may be due to the grey literature and hand searched references not being restricted by date as had the electronic searches. This highlights the differences in approach required in searching the electronic and grey literature, and the challenges inherent in designing an inclusive search strategy. In view of this, the grey literature search in particular may not have captured all relevant articles; however, every attempt was made to minimise the potential of ‘missed’ literature by developing the search strategy with input from experts in the field of podiatry and foot health care, and guided by a specialist in designing literature search strategies.

The reader needs also be aware that while the GRADE system is widely adopted by medical researchers, it does have its limitations. Often manuscripts retrieved from the grey literature, such as observational studies, narratives and clinical expert opinion, tend to be classified as being low methodological quality; however in the absence of any other more suitable system it was decided to use GRADE as “*the extensive user testing done on the GRADE system could be one factor that has contributed to its popularity among guideline developers*” [35].

Lastly, quality of evidence was determined by methodological assessment of the publications in accordance with the AGREE II consortium and interpretation of cited evidence [27] through the Cochrane’s recommended approach for grading the quality of evidence (GRADE) [31]. We found difficulty in conducting these assessments on all publications due to the high proportion of narrative reviews and publications based on consensus /expert opinion that were included. Often, there was circularity of evidence and recommendations in such publications tended to have very little new evidence included. We attempted to reduce the biases that may have been introduced due to the subjective nature in these assessments by ensuring all reviewers met three times during the assessment period to discuss controversies and arrive at consensus for the final scores. We also ensured consistency in scoring by allocating publications for assessment and scoring between the three reviewers (KE, CB, AB). CB and AB independently scored 83 publications and KE independently scored 40 of each of AB and CBs publications.

### Recommendations

1. Gathering evidence for the effectiveness of core podiatry (corns, callus and nail care) and the effectiveness of podiatric surgery should be a high priority for the podiatry and foot and ankle research communities.
2. Professional bodies and the foot and ankle research communities should have a coordinated podiatry (or equivalent foot health care clinicians in countries where podiatry does not exist) representation on key policy and national and international guideline development committees that have a focus on foot and ankle health and management.
3. Professional bodies and the foot and ankle research communities should have coordinated collaboration for collection of outcomes data and evidence that demonstrates the value and impact of podiatry and foot health care on patients.
4. Future guidelines, standards of care and recommendations should follow a recognised framework (e.g. AGREE II) and use one validated standardised system for grading evidence (i.e. GRADE) for podiatry and foot health care or alternatively have a system that will enable comparability between grading systems (as attempted to do in this paper).

## Conclusion

It is clear that evidence needs to be accelerated and in place to support the future of the podiatry and foot health clinical workforce. In this systematic review, we have found that the field has an emergent evidence base that is becoming more established year on year. We have also discovered small but relevant numbers of high level evidence for the effectiveness of podiatry and foot health management especially concerning assessment for monitoring and prevention of serious foot complications associated with chronic conditions. Where levels of evidence have been given in high quality guidelines, standards of care or recommendations, they also tend to be strong-moderate quality such that further strategically prioritised research, if performed, is likely to have an important impact in the field.

The results from this study will help inform the stakeholders and users of podiatry services such as podiatry clinicians, clinical guideline developers or policymakers on evidence for foot health management in their decision making.

## Additional files

Additional file 1: Appendix A. Electronic search strategy: an example using Embase. (DOCX 84 kb)

Additional file 2: Appendix B. Scoring of included papers against AGREE II criteria for quality assessment. (DOCX 140 kb)

Additional file 3: Appendix C. Types of evidence within the included publications. (DOCX 100 kb)

Additional file 4: Appendix D. Levels of evidence where reported from included studies. (DOCX 41 kb)

Additional file 5: Appendix E. Levels of Evidence for Recommendations concerning Foot Health Management. (DOCX 28 kb)

## Abbreviations

AGREE: Appraisal of guidelines for research and evaluation
GRADE: Grading of recommendations, assessment development and evaluation system
PICO: Patient, problem or population, intervention, comparison, control or comparator, outcome
PRISMA: Preferred reporting items for systematic reviews and meta- analyses
RCT: Randomised controlled trial
UK: United Kingdom
