# Supplementary material for: Evidence for current recommendations concerning the management of foot health for people with chronic long-term conditions: a systematic review
Source: J Foot Ankle Res. 2017 Nov 22;10:51. doi: 10.1186/s13047-017-0232-3 (PMC5700544; doi:10.1186/s13047-017-0232-3)
Supplement: Supplementary file 1 — Electronic search strategy: an example using Embase. (DOCX 84 kb) [file 13047_2017_232_MOESM1_ESM.docx]

**Supplementary file 1: Podiatry evidence systematic review**

## **Appendix A:** Electronic search strategy: an example using Embase

Embase 1974 to 2016 September 15

SEARCH DATE: 16/09/16

1 exp practice guideline/

2 Health Planning Guidelines/

3 "guideline*".m_titl.

4 (practice adj3 parameter*).ti,ab.

5 Clinical Protocols/

6 guidance.ti,ab.

7 "care pathway*".ti,ab.

8 Critical Pathways/

9 (clinical adj3 pathway*).ti,ab.

10 Algorithms/

11 (consensus adj2 development).ti,ab.

12 "quality and outcomes framework*".ti,ab.

13 OR/ 1-12

14 foot.ti,ab.

15 feet.ti,ab.

16 podiatr*.ti,ab.

17 Podiatry/

18 OR/ 14-17

19 chronic*.hw.

20 ((chronic* or persistent or long* term or ongoing) adj (disease* or disab* or ill* or condition* or health condition* or medical condition*)).tw.

21 chronic fatigue syndrome.tw.

22 degenerative disease*.tw.

23 long term care/

24 long* term care.tw.

25 exp neurodegenerative diseases/

26 (neurodegenerative or Huntington* disease or Parkinson* disease or amyotrophic lateral sclerosis or motor neuron disease).tw.

27 exp multiple sclerosis/

28 multiple sclerosis.tw.

29 exp arthritis/

30 exp lung diseases obstructive/

31 (obstructive lung disease* or obstructive pulmonary disease* or asthma or bronchitis).tw.

32 exp emphysema/

33 exp pulmonary emphysema/

34 emphysema.tw.

35 chronic disease/

36 exp diabetes mellitus/

37 (diabetes or diabetic).tw.

38 exp hypertension/

39 (hypertension or high blood pressure).tw.

40 exp cerebrovascular disorders/

41 (cerebrovascular disease* or cerebrovascular disorder* or brain ischemia or cerebral infarction or carotid artery disease* or stroke).tw.

42 exp myocardial ischemia/

43 (myocardial ischemia or angina pectoris or coronary disease* or coronary artery disease* or myocardial infarction).tw.

44 exp heart failure/

45 (heart failure or heart disease*).tw.

46 exp colonic diseases/

47 (colonic disease* or colitis or irritable bowel syndrome).tw.

48 exp hiv/

50 (hiv infect* or hiv disease*).tw.

51 exp osteoporosis/

52 osteoporosis.tw.

53 fibromyalgia/

54 fibromyalgia*.tw.

55 exp neoplasms/

56 (cancer* or oncolog* or neoplasm* or carcinom* or tumo?r* or malignan*).tw.

57 exp mental disorders/

58 depression/

59 ((mental* or psychiatr* or psychological*) adj (ill* or disorder* or disease* or distress* or disab* or problem* or health* or patient* or treatment)).tw.

60 ((personality or mood or dysthymic or cognit* or anxiety or stress or eating or adjustment or reactive or somatoform or conversion or behavior or perception or psycho* or impulse control or development*) adj disorder*).tw.

61 (psychos#s or psychotic* or paranoi* or schizo* or neuros#s or neurotic* or delusion* or depression or depressive or bipolar or mania or manic or obsessi* or compulsi* or panic or phobic or phobia or anorexia or bulimia or neurastheni* or dissociative or affective or borderline or narcissis* or suicid* or self injur* or self harm or dementia or Alzheimer*).tw.

62 exp asthma/

63 exp pulmonary disease chronic obstructive/

64 (chronic adj2 obstructive adj2 pulmonary).tw.

65 exp thyroid diseases/

66 exp hyperlipidemia/

67 exp substance-related disorders/ or exp substance abuse/

68 (arthritis or osteoarthritis or rheumat*).tw.

69 exp Gout/

70 gout.ti,ab.

71 Arthritis, Psoriatic/

72 "psoria* arthritis".ti,ab.

73 Spondylitis, Ankylosing/

74 (Ankyl* adj2 spondyl*).ti,ab.

75 Colitis, Ulcerative/

76 (colitis adj2 ulcerative).ti,ab.

77 (Colitic adj2 arthritis).ti,ab.

78 Scleroderma, Diffuse/

79 Scleroderma*.ti,ab.

80 OR/ 20-79

81 14 and 19 and 80

82 limit 81 to (english language and yr="2000 -Current")
