# Supplementary material for: Evidence for current recommendations concerning the management of foot health for people with chronic long-term conditions: a systematic review
Source: J Foot Ankle Res. 2017 Nov 22;10:51. doi: 10.1186/s13047-017-0232-3 (PMC5700544; doi:10.1186/s13047-017-0232-3)
Supplement: Supplementary file 2 — Scoring of included papers against AGREE II criteria for quality assessment. (DOCX 140 kb) [file 13047_2017_232_MOESM2_ESM.docx]

**Supplementary file 2: Podiatry evidence systematic review**

## **Appendix B:** Scoring of Included Papers Against AGREE (II) Criteria for Quality Assessment

| **URN** | **Author(s)** | **AGREE II (2010) domains** | | | | | | | **I would recommend** |
| --- | --- | --- | --- | --- | --- | --- | --- | --- | --- |
|  |  | **1** | **2** | **3** | **4** | **5** | **6** | **Overall** |  |
| H1 | ACR/EULAR | 21 | 21 | 21 | 21 | 17 | 14 | 5 | yes with modifications |
| H87 | ADA | 8 | 7 | 8 | 9 | 8 | 2 | 1 | no |
| G54 | AHRQ | 12 | 12 | 21 | 12 | 12 | 4 | 4 | yes |
| G57 | AHRQ | 12 | 12 | 21 | 12 | 12 | 4 | 4 | yes |
| G58 | AHRQ | 12 | 12 | 21 | 12 | 12 | 4 | 4 | yes |
| G61 | AHRQ | 12 | 12 | 21 | 12 | 12 | 4 | 4 | yes |
| G65 | AHRQ | 12 | 12 | 21 | 12 | 12 | 4 | 4 | yes |
| G69 | AHRQ | 12 | 12 | 21 | 12 | 12 | 4 | 4 | yes |
| G73 | AHRQ | 12 | 12 | 21 | 12 | 12 | 4 | 4 | yes |
| G55 | AHRQ | 21 | 21 | 46 | 21 | 6 | 14 | 7 | yes |
| G56 | AHRQ | 21 | 12 | 46 | 21 | 14 | 14 | 7 | yes |
| G59 | AHRQ | 21 | 15 | 47 | 21 | 8 | 14 | 7 | yes |
| G60 | AHRQ | 21 | 12 | 38 | 19 | 11 | 14 | 6 | yes |
| G63 | AHRQ | 21 | 13 | 49 | 21 | 14 | 14 | 7 | yes |
| G64 | AHRQ | 14 | 12 | 47 | 21 | 9 | 14 | 6 | yes |
| G71 | AHRQ | 21 | 10 | 48 | 21 | 19 | 14 | 7 | yes |
| E84 | Aiello.A., Anichini.R., Brocco.E., Caravaggi.C., Chiavetta.A., Cioni.R., Da Ros.R., et al | 20 | 18 | 16 | 18 | 10 | 2 | 6 | yes |
| G76 | Ameen. M., Lear.J.T., Madan.V., Mohd Mustapa.M.F., Richardson.M. | 21 | 15 | 53 | 21 | 9 | / | 6 | yes |
| H114 | American Diabetes Association | 12 | 12 | 18 | 12 | 12 | 2 | 3 | no |
| H115 | American Diabetes Association | 18 | 12 | 18 | 12 | 12 | 2 | 3 | no |
| H18 | American Diabetes Association | 21 | 21 | 21 | 21 | 21 | 14 | 6 | yes |
| E7 | American Diabetes Association | 20 | 13 | 25 | 11 | 6 | 5 | 3 | no |
| H43 | American Diabetes Association | 13 | 5 | / | 8 | 6 | / | 2 | no |
| H45 | Amod.A., Ascott-Evans.B.H., Berg.G.I., Blom.D.J., Brown.S.L., Carrihill.M.M., Dave.J.A., Distiller.L.A., et al | 17 | 9 | 21 | 10 | 12 | 3 | 4 | yes with modifications |
| E36 | Anondyne therapy, Bristol Myers-Squibb & Diabteics United | 15 | / | / | 14 | 10 | 14 | 3 | 1 |
| G119 | ARMA | 21 | 21 | 32 | 21 | 24 | 7 | 6 | yes |
| H80 | ARMA | 12 | 13 | / | 12 | 7 | 4 | 3 | no |
| I3 | Baker et al | 21 | 21 | 48 | 21 | 21 | 14 | 7 | yes |
| E68 | Baker.N and Kenny,C. | 15 | 3 | 18 | 12 | 4 | 4 | 3 | no (not guideline) |
| I4 | Bakker.K., Apelqvist.J., Lipsky.B.A., van Netten.J.J., and International working group on the diabetic foot | 19 | 11 | 13 | / | / | 7 | 3 | / |
| E66 | Bakker.K., Apelqvist.J., Schaper.N.C., on behalf of the International working group on the diabetic foot editorial board | 12 | 12 | / | 12 | 6 | 8 | 2 | no |
| E57 | Benbow.S. | 21 | / | / | 18 | 10 | 14 | / | / |
| E79 | Berendt.A.R., Peters.E.J.G., Bakker.K., Embil.J.M., Eneroth.M., Hinchliffe.R.J., Jeffcoate.W.J., Lipsky.B.A., Senneville.E., The.J., and Valk.G.D. | 17 | / | / | 12 | / | 10 | 3 | 1 |
| E21 | Blanes.J.I., Representatives of Spanish society of surgeons (ACS), Representatives of Spanish society of angiology and vascular surgery (SEACV), Representatives of Spanish society of emergency medicine (SEMES), Spanish internal medicine society (SEMI); Representatives of Spanish society critical care medicine and coronary unit(SEMICYUC), Representatives of Spanish society of chemotherapy (SEQ) | 20 | 14 | 16 | 18 | 7 | 4 | 4 | yes with modifications |
| E64 | Boike.A.M., and Hall.J.O. | 9 | 7 | 8 | 9 | 8 | 2 | 1 | no |
| H86 | Boulton.A.J., Gries.F.A., Jervell.J.A. | 15 | 11 | / | 10 | 6 | 4 | 3 | no |
| E34 | Bowering & Embil | 13 | 6 | 20 | 18 | 9 | 6 | 4 | yes with modifications |
| E14 | Bowring.B., and Chockalingham.N. | 21 | 7 | 25 | 3 | 8 | 14 | 4 | no |
| E89 | Braun.L., Kim.P.J., Peters.E.J., Lavery.L.A., and Wound healing society. | 19 | 5 | 26 | 14 | 4 | 6 | 5 | yes |
| E15 | Bristow.I.R., de Berker.D.A.R., Acland.K.M., Turner.R.J., and Bowling.J. | 21 | 7 | 17 | 17 | 7 | 14 | 3 | yes with modifications |
| I8 | Bus.S.A., Armstrong.D.G., van Deuren.R.W., Lewis.J.E., Caravaggi.P.R., and International working group on the diabetic foot | / | / | 14 | 18 | / | 7 | 3 | / |
| E80 | Bus.S.A., Valk.G.D., van Deursen.R.W., Armstrong.D.G., Caravaggi.C., Hlavacek.P., Bakker.K., and Cavanagh.P.R. | 10 | 10 | 16 | 12 | 8 | 12 | 2 | no |
| I9 | Bus.S.A., van Netten.J.J., Lavery.L.A.,Monteiro-Soares.M., Rasmussen.A., Jubiz.Y., Price.P.E., and International working group on the diabetic foot | / | / | 14 | 18 | / | 7 | 3 | / |
| E47 | Campbell.L.V., Graham.A.R., Kidd.R.M., Molloy.H.F., O'Rourke.S.R., and Colagiuri.S. | 9 | 7 | 8 | 6 | 7 | 2 | 2 | no |
| H106 | Canadian Diabetes Association | 18 | 15 | 56 | 21 | 22 | 14 | 7 | yes |
| H76 | Canadian Diabetes Association | 14 | 13 | 22 | 17 | 17 | 8 | 4 | yes with modifications |
| H6 | Caribbean Health Council | 21 | 13 | 12 | 19 | 4 | 2 | 4 | yes |
| H84 | Clark.M.J.Jr., Sterrett.J.J., Carson.D.S. | 12 | 12 | 18 | 18 | 10 | 6 | 3 | no |
| E43 | Collieris | 5 | 7 | 8 | 3 | 4 | 2 | 1 | no |
| G121 | Combe.B., Landewe.R., Lukas.C., Bolosiu.H.D., Breedveld.F., Dougados.M., Emery.P., Ferraccioli.G., Hazes.J.M.W., Klareskog.L., Machold.K., Martin-Mola.E., Nielsen.H., Silman.A., Smolen.J., Yazici.H. | 21 | 12 | 46 | 21 | 8 | 4 | 6 | yes |
| G108 | Dasgupta.B., Borg.F.A., Hassan.N., Barraclough.K., Bourke.B., Fulcher.J., Hollywood.J., Hutchings.A., Kyle.V., Nott.J., et al | 21 | 9 | 19 | 19 | 4 | 8 | 4 | yes |
| H77 | Day | 12 | 5 | / | / | / | / | 1 | no |
| H105 | Diabetes UK | 18 | 9 | 18 | 12 | 12 | 2 | 3 | no |
| G95 | DoH | 21 | 14 | 22 | 19 | 6 | 2 | 4 | yes with modifications |
| G111 | DoHA | 21 | 21 | 53 | 21 | 12 | 10 | 7 | yes |
| G21 | Dorresteijn.J.A., Kriegsman.D.M., Assendelft.W.J., Valk.G.D. | 21 | 21 | 21 | 21 | 21 | 14 | 7 | yes |
| H92 | Edmonds.M. | 13 | 10 | / | 13 | / | 10 | 3 | no |
| E3 | Evans | 14 | 9 | 0 (?) | 11 | 9 | 12 | 3 | no |
| E37 | Evans.A., and Philips.P. | 8 | 2 | 8 | 5 | 4 | 2 | 1 | no |
| E58 | Forestier.R., Andre-Vert.J., Guillez.P., Coudeyre.E., Lefevre-Colau.M.M., Combe.B., Mayoux-Benhamou,M.A. | 21 | 18 | 51 | 21 | 8 | 7 | 6 | yes |
| I16 | Frankel.A., Kazempour-Ardebili.S., Bedi.R., Chowdhury.T.A., et al | 11 | 7 | 14 | 21 | / | 14 | 4 | / |
| H61 | Frykberg.R.G. | 6 | 4 | / | 9 | / | / | 1 | no |
| E27 | Frykberg.R.G., Zgonis.T., Armstrong.D.G., Driver.V.R., Giurini.J.M., Kravitz.S.R., Landsman.A.S., et al | 21 | 5 | 26 | 19 | 4 | 8 | 4 | yes with modifications |
| H118 | Frykberg.R.G., Zgonis.T., Armstrong.D.G., Driver.V.R., Giurini.J.M., Kravitz.S.R., Landsman.A.S., et al | 15 | 7 | / | 8 | 6 | / | 2 | no |
| G106 | Gain | 10 | 10 | 16 | 18 | 10 | 2 | 1 | no |
| I17 | Game.F.L., Apelqvist.J., Attinger.C., Hartemann.A., Hinchliffe.R.J., Londahl.M., Price.P.E., Jeffcoate.W.J., International working group on the diabetic foot | 19 | / | 7 | 18 | / | 7 | 3 | / |
| E59 | Gossec et al | 15 | 12 | 18 | 18 | 8 | 4 | 1 | no |
| G127 | Guidance: National Service Framework: Diabetes | 21 | 11 | / | 19 | 6 | / | 4 | yes, with modifications |
| H34 | Hinchcliffe.R.J., Brownrigg.J.R.W., Apelqvist.J., Boyko.E.J., Fitridge.R., Mills.J.L., Reekers.J., et al | 21 | 12 | 49 | 21 | 4 | 2 | 7 | yes |
| I20 | Hinchliffe.R.J., Brownrigg.J.R., Apelqvist.J., Boyko.E.J.Fitidge.R., Mills.J.L., Reekers.J., Shearman.C.P., Zierler.R.E., Schaper.N.C., International working group on the diabetic foot | 16 | / | 11 | 18 | / | / | 3 | / |
| I21 | Hingorani.A., LaMuraglia.G.N., Henke.P., Meissner.M.H., Loretz.L., Zinszer.K.M., Driver.V.R., Frykberg.R., Carman.T.L., Marston.W., Mills.J.L.Sr., Murad.M.H. | 20 | 6 | 25 | 18 | / | 14 | 5 | / |
| I32 | Huang.E.T., Mansouri., Murad.M.H., Joseph.W.S., Strauss.M.B., et al | 21 | 21 | 50 | 21 | 9 | 14 | 7 | yes |
| E16 | Hutchinson.A.., McIntosh.A., Feder.G.,Home.P.D.,Young.R. | 18 | 17 | 0 | 18 | 12 | 12 | 4 | yes with modifications (summary doc) |
| G89 | International diabetes federation | 21 | 21 | 21 | 21 | 21 | 14 | 7 | yes |
| G93 | International diabetes federation | 12 | 12 | 21 | 12 | 12 | 4 | 4 | yes |
| I34 | Isei.T., Abe.M., Nakanishi.T., Matsuo.K., Yamasaki.O., Asana.Y., Ishii.T., et al | 21 | 7 | 37 | 19 | 4 | 8 | 5 | yes, with modifications |
| H41 | Jirsch.A.T., Haskal.Z.J., Hertzer.N.R., Bakal.C.W., Creager.M.A., Halperin.J.L., Hirazka.L.F., Murphy.W.R.C., Olin.J.W., et al | 21 | 17 | 52 | 21 | 4 | 14 | 6 | yes |
| I36 | Lavery.L.A., Davis.K.E., Berriman.S.J., Braun.L., Nichols.A., Kim.R.J., Margolis.D., Peters.E.J., Attinger.C | 21 | 12 | / | 19 | / | / | / | / |
| I37 | Lipsky.B.A., Aragon-Sanchez.J., Diggle.M., Embil.J., Kono.S., Lavery.L., et al | 21 | 9 | 34 | 13 | 14 | 5 | 5 | yes |
| E2 | Lipsky.B.A., Berendt.A.R., Cornia.P.B., Pile.J.C., Peters.E.J.G., Armstrong.D.G., Gunner Deery.H., Embil.J.M., Joseph.W.S., Karchmer.A.W., Pinzur.M.S., and Senneville.E. | 21 | 10 | 53 | 21 | 11 | 14 | 6 | yes |
| H23 | Lipsky.B.A., International consensus group on diagnosing and treating the infected diabetic foot | 21 | / | / | 21 | 4 | / | 5 | 1 |
| E78 | Lipsky.B.A., Peters.E.J.G., Berendt.A.R., Senneville.E., Bakker.K., Embil.J.M., Lavery.L.A., Urbancic-Rovan.V., and Jeffcoate.W.J. | 15 | / | / | 11 | 4 | 10 | 3 | 1 |
| G102 | Machado et al | 21 | 10 | 47 | 21 | 4 | 8 | 6 | yes |
| E48 | Malik.R.A. | 9 | 7 | 8 | 9 | 6 | 2 | 1 | no |
| H85 | Massachusetts Department of Public Health | 16 | 14 | 8 | 18 | 14 | 5 | 4 | yes with modifications |
| E69 | Mayfield.J.A., Reiber.G.F., Sanders.L.J., Janisse.D., and Pogach.L.M. | 9 | 7 | 8 | 9 | 8 | 2 | 1 | no |
| E71 | Mayfield.J.A., Reiber.G.F., Sanders.L.J., Janisse.D., and Pogach.L.M. | 9 | 7 | 8 | 9 | 8 | 2 | 1 | no |
| H71 | McInnes.A., Jeffcoate.W., Vileikyte.L., Game.F., Lucas.K., Higson.N., Stuart.L., Church.A., Scanlan.J., Anders.J. | 15 | 15 | 21 | 15 | 18 | 14 | 5 | yes |
| H113 | Meltzer et al | 16 | 13 | 32 | 17 | 16 | 8 | 5 | yes with modifications |
|  | Ministerio de Sanidad y Consum | 21 | 19 | 41 | 21 | / | 4 | 7 | yes |
| E50 | Moncada.L. van Voast | 18 | 5 | 28 | 19 | 10 | 1 | 6 | yes |
| E60 | Murphy.J., Best.C., Cavan.D., and Kerr.D. | 9 | 5 | 8 | 9 | 6 | 2 | 1 | no |
| E87 | Nather.A., Siok Bee. Keng Lin.W., Xin-Bei Valerie.C., Liang.Shen., et al | / | / | / | / | / | / | / | / |
| H81 | National Diabetes Education Program | 14 | 11 | / | 10 | 12 | / | 4 | yes, with modifications |
| H96 | National Minimum Skills Framework | 14 | 13 | / | 8 | / | / | 3 | no |
| G112 | NHS QI | 21 | 12 | 41 | 21 | 4 | / | 7 | yes |
| G101 | NHS Scotland | 12 | 12 | 18 | 12 | 12 | 6 | 4 | no |
| G1 | NICE | 21 | 21 | 21 | 21 | 21 | 14 | 7 | yes |
| G13 | NICE | 21 | 21 | 21 | 21 | 21 | 14 | 7 | yes |
| G14 | NICE | 21 | 21 | 21 | 21 | 21 | 14 | 7 | yes |
| G2 | NICE | 21 | 21 | 21 | 21 | 21 | 14 | 7 | yes |
| G33 | NICE | 21 | 21 | 21 | 21 | 21 | 14 | 7 | yes |
| G4 | NICE | 21 | 21 | / | 21 | 21 | 14 | 7 | yes |
| G5 | NICE | 21 | 21 | 21 | 21 | 21 | 14 | 7 | yes |
| G6 | NICE | 21 | 21 | 21 | 21 | 21 | 14 | 7 | yes |
| G9 | NICE | 21 | 21 | 21 | 21 | 21 | 14 | 7 | yes |
| G12 | NICE | 21 | / | / | / | / | / | / | / |
| G3 | NICE | 21 | 7 | / | 17 | 4 | 8 | 7 | yes |
| G8 | NICE | 21 | 21 | / | / | / | / | 7 | yes |
|  | NICE CKS (Achilles Tendinopathy) | 21 | / | / | 21 | / | / | 7 | yes |
|  | NICE CKS (Bunions) | 21 | / | / | 21 | / | / | 7 | yes |
|  | NICE CKS (Diabetes - type 2) | 21 | / | / | 21 | / | / | 7 | yes |
|  | NICE CKS (Diabetes -type 1) | 21 | / | / | 21 | / | / | 7 | yes |
|  | NICE CKS (Fungal nail infection) | 21 | / | / | 21 | / | / | 7 | yes |
|  | NICE CKS (Gout) | 21 | / | / | 21 | / | / | 7 | yes |
|  | NICE CKS (Morton's neuroma) | 21 | / | / | 21 | / | / | 7 | yes |
|  | NICE CKS (Osteoarthritis) | 21 | / | / | 21 | / | / | 7 | yes |
|  | NICE CKS (Peripheral arterial disease) | 21 | / | / | 21 | / | / | 7 | yes |
|  | NICE CKS (Plantar fasciitis) | 21 | / | / | 21 | / | / | 7 | yes |
|  | NICE CKS (Rheumatoid arthritis) | 21 | / | / | 21 | / | / | 7 | yes |
|  | NICE CKS (Stroke and TIA) | 21 | / | / | 21 | / | / | 7 | yes |
| G17 | NICE Quality & productivity | 18 | 10 | 20 | 16 | 9 | 2 | 4 | yes with modifications |
| H74 | Norgren.L., Hiatt.W.R., Dormandy.J.A., Nehler.M.R., Harris.K.A., Fowkes.F.G., Rutherford.R.B., TASC II working group | 12 | 9 | 23 | 21 | 8 | 12 | 4 | yes with modifications |
| G84 | NWpodiatryCEG | 21 | 8 | 15 | 16 | 7 | / | 4 | 1 |
| H32 | Orsted.H.L., Searles.G.E., Trowell.H., Shapera.L., Miller.P., Rahman.J. | 21 | / | / | 19 | 7 | 14 | 7 | yes |
| G29 | Ortegon.M.M., Redekop.W.K., Niessen.L.W. | 18 | 9 | 26 | 12 | 16 | 6 | 4 | yes |
| H119 | Patel.V., and Morrissey.J. | 5 | 5 | / | / | 8 | / | 1 | no |
| E23 | Patout.C.A., Birke.J.A., Wilbright.W.A., Coleman.W.C., and Mathews.R.E. | 21 | 5 | 10 | 16 | 4 | 2 | 3 | no |
| H59 | Pinzur.M.S., Slovenkai.M.P., Trepman.E., Shields.N.N., Diabetes committee of American Orthopaedic foot and ankle society | 9 | 6 | / | 10 | 6 | / | 2 | no |
| G88 | PSA working group | 21 | 10 | 13 | 21 | 4 | 2 | 4 | yes with modifications |
| G109 | RACGPs | 12 | 12 | 18 | 12 | 12 | 6 | 4 | no |
| G82 | RACGPs | 20 | 18 | 16 | 12 | 18 | 12 | 6 | yes |
| H7 | Redmon.B., Caccami.D., Flavin.P., Michels.R., O'Connor.P., Roberts.J., Smith.S., Sperl-Hillen.J. | 21 | 14 | 31 | 21 | 12 | 2 | 6 | yes |
| H27 | Registered nurses' association | 16 | 14 | 50 | 16 | 19 | 8 | 6 | yes |
| H28 | Registered nurses' association | 19 | 14 | 50 | 14 | 19 | 5 | 6 | yes |
| E41 | Rheeder et al | 21 | 19 | 51 | 21 | 16 | 10 | 7 | yes |
| E73 | Robbins.J.R., Nicklas.B.J., and Augustine.S. | 9 | 7 | 8 | 9 | 8 | 2 | 1 | no |
| E83 | Rymaszewski.L.A., Sharma.S., McGill.P.E., Murdoch.A., Freeman.S, and Loh.T. | / | / | / | / | / | / | / | / |
| G103 | Salem (SCP) | 17 | 11 | 15 | 15 | 4 | 5 | 4 | yes with modifications |
| H31 | Schaper.N.C., Andros.G., Apelqvist.J., Bakker.K., Lammer.J., Lepantalo.M., Mills.J.L., Reekers.J., Shearman.C.P., Zierler.R.E., Hinchliffe.R.J., International working group on diabetic foot | 21 | / | / | 21 | 4 | 4 | / | / |
| H30 | Setacci.C., Ricco.J.B., European Society for Vascular Surgery | 21 | / | / | / | / | / | / | / |
| G122 | SIGN | 20 | 21 | 21 | 21 | 21 | 10 | 4 | no |
| G77 | SIGN | 21 | 21 | 21 | 21 | 21 | 14 | 7 | yes |
| G98 | SIGN | 21 | 21 | 21 | 21 | 21 | 14 | 4 | yes |
| G100 | SIGN | 21 | 21 | 56 | 21 | 28 | / | 7 | yes |
| H111 | SIGN 116 | 21 | 21 | 56 | 21 | 28 | 14 | 7 | yes |
| H112 | SIGN 55 | 21 | 21 | 56 | 21 | 28 | 14 | 7 | yes |
| G30 | Singh.N., Armstrong.D.C., and Lipsky.B.A. | 9 | 11 | 18 | 12 | 12 | 2 | 4 | yes |
| E33 | Soliman.A., and Brogan.M. | 8 | 2 | 8 | 5 | 4 | 2 | 1 | no |
| E42 | Steed.D.L., Attinger.C., Brem.H., Colaizzi.T., Crossland.M., et al | 20 | 13 | 32 | 7 | 4 | 1 | 4 | yes with modifications |
| H48 | Steed.D.L., Attinger.C.,. Colaizzi.T., Crossland.M., Franz.M., Harkless.L., Johnson.A., et al | 12 | 8 | 24 | 12 | 4 | / | 4 | yes, with modifications |
| H3 | The National Board of Health and Welfare | 17 | 15 | / | 16 | 16 | / | 4 | yes, with modifications |
| E31 | Thomas.J.L., Christensen.J.C., Kravitx.S.R., Mendicino.R.W., Schuberth.J.M., Vanore.J.V., et al | 14 | 12 | revision | 14 | 6 | 12 | 4 | yes with modifcations |
| H62 | Thomson.F.J., Veves.A., Ashe.H., Knowles.E.A., Gem.J., Walker.M.G., Hirst.P., Boulton.A.J.M | 8 | 5 | / | 7 | / | / | 1 | no |
| H91 | Tovey.F.I. | 6 | 6 | 8 | 9 | 7 | 2 | 1 | no |
| H66 | VA/DoD | 18 | 17 | 46 | 18 | 28 | / | 6 | yes |
| 150 | Walker, C., Bunch, F.T., Cavros.N.G., and Dippel.E.J. | 16 | 8 | 14 | 14 | 4 | 6 | 4 | no |
| 151 | Weinstein, et al | 21 | 14 | 48 | 21 | 4 | 14 | 6 | yes, with modifications |
| H57 | Whitney et al | 12 | 14 | 26 | 12 | 6 | / | 5 | yes, with modifications |
| H72 | WHO | 13 | 6 | / | 10 | 6 | / | 2 | no |
| E40 | Williams.A.E., Davies.S., Graham.A., Dagg.A., Longrigg.K., Lyons.C., Bowen.C. | 18 | 18 | 25 | 16 | 8 | 8 | 5 | yes |
| G116 | World Health Organisation | 21 | / | / | 21 | 11 | / | 7 | yes |
| E22 | Wraight et al | 20 | 18 | 20 | 9 | 8 | 4 | 4 | yes with modifications |
| H93 | Wukich.D.K., Armstrong.D.G., Attinger.C.E., Boulton.A.J., Burns.P.R., Frykbery.R.G., Hellman.R., Kim.P.J., Lipsky.B.A., et al | 13 | 10 | / | 8 | 5 | 7 | 2 | no |
| G124 | Zhang.W., Doherty.M., Pascual.E., Bardin.T., Barskova.V., Conaghan.P., Gerster.J., Jacobs.J., Leeb.B., Liote.F., et al | 21 | 21 | 21 | 21 | 21 | 14 | 4 | yes |
| G123 | Zhang.W., Doherty.M., Pascual.E., Barskova.V., Conaghan.P., Gerster.J., Jacobs.J., Leeb.B., et al | 21 | 21 | 21 | 21 | 21 | 14 | 4 | yes |
| H1 | ACR/EULAR | 21 | 21 | 21 | 21 | 17 | 14 | 5 | yes with modifications |
