# Supplementary material for: Evidence for current recommendations concerning the management of foot health for people with chronic long-term conditions: a systematic review
Source: J Foot Ankle Res. 2017 Nov 22;10:51. doi: 10.1186/s13047-017-0232-3 (PMC5700544; doi:10.1186/s13047-017-0232-3)
Supplement: Supplementary file 3 — Types of evidence within the included publications. (DOCX 100 kb) [file 13047_2017_232_MOESM3_ESM.docx]

**Supplementary file 3: Podiatry evidence systematic review**

## **Appendix C:** Types of evidence within the Included Publications

| **AGREE** | **Core podiatry**  **(A)** | **Foot ulcer care**  **(B)** | **Foot health education**  **(C)** | **Preventative foot care & assessments**  **(D)** | **Preventative care advancements**  **(E)** | **Clinical Interventions**  **(F)** | **Podiatric surgery**  **(G)** | **Other**  **(H)** | **Pain medication**  **(I)** | **Antibiotics**  **(J)** | **Orthopaedic surgery**  **(K)** | **Antifungals**  **(L)** | **Footwear assessments**  **(M)** |
| --- | --- | --- | --- | --- | --- | --- | --- | --- | --- | --- | --- | --- | --- |
| **1** | N=6  **[6]** | R/C=1  N=12  **[13]** | N=4  **[4]** | R/C=1  N=12  **[13]** | R/C=1  N=3  **[4]** | LoE=1  N=3  **[4]** | N=2  **[2]** | N=1  **[1]** | **[0]** | N=1  **[1]** | **[0]** | **[0]** | N=2  **[2]** |
| **2** | R/C=1  N=1  **[2]** | R/C=4  N=3  **[7]** | R/C=2  N=4  **[6]** | R/C=3  N=3  **[6]** | R/C=1  **[1]** | R/C=3  N=2  **[5]** | R/C=1  **[1]** | R/C=1  **[1]** | **[0]** | R/C=1  **[1]** | **[0]** | **[0]** | **[0]** |
| **3** | LoE=1  R/C=2  N=3  **[6]** | LoE=4  R/C=3  N=4  **[11]** | LoE=1  R/C=3  N=5  **[9]** | LoE=1  R/C=4  N=7  **[12]** | R/C=1  N=1  **[2]** | LoE=3  R/C=1  N=3  **[7]** | LoE=1  R/C=2  **[3]** | **[0]** | **[0]** | R/C=4  **[4]** | R/C=1  **[1]** | **[0]** | R/C=1  N=3  **[4]** |
| **4** | LoE=3  R/C=2  N=4  **[9]** | LoE=9  R/C=3  N=9  **[21]** | LoE=8  R/C=3  N=12  **[23]** | LoE=13  R/C=3  N=18  **[34]** | LoE=6  R/C=1  N=6  **[13]** | LoE=11  R/C=2  N=10  **[23]** | LoE=2  R/C=1  N=1  **[4]** | **[0]** | LoE=1  R/C=1  N=1  **[3]** | LoE=6  R/C=2  N=4  **[12]** | R/C=1  N=1  **[2]** | N=1  **[1]** | LoE=3  R/C=2  N=4  **[9]** |
| **5** | LoE=1  R/C=2  **[3]** | LoE=5  R/C=1  **[6]** | R/C=1  **[1]** | LoE=4  R/C=3  **[7]** | LoE=2  R/C=2  **[4]** | LoE=3  R/C=1  **[4]** | LoE=1  **[1]** | N=1  **[1]** | **[0]** | LoE=4  R/C=1  **[5]** | LoE=1  **[1]** | LoE=1  **[1]** | R/C=1  **[1]** |
| **6** | LoE=4  R/C=2  N=1  **[7]** | LoE=6  R/C=1  **[7]** | LoE=7  R/C=2  N=2  **[11]** | LoE=8  R/C=2  N=1  **[11]** | LoE=5  R/C=2  N=1  **[8]** | LoE=7  R/C=1  **[8]** | R/C=1  **[1]** | R/C=1  **[1]** | LoE=1  R/C=1  **[2]** | LoE=1  **[1]** | LoE=1  R/C=1  **[2]** | LoE=1  N=1  **[2]** | LoE=5  R/C=1  **[6]** |
| **7** | LoE=2  N=1  **[3]** | LoE=5  N=4  **[9]** | LoE=8  N=8  **[16]** | LoE=11  N=22  **[33]** | LoE=5  N=7  **[12]** | LoE=7  N=17  **[24]** | N=2  **[2]** | N=1  **[1]** | LoE=1  N=1  **[2]** | LoE=3  N=3  **[6]** | **[0]** | N=1  **[1]** | LoE=2  N=1  **[3]** |
| **/** | N=1  **[1]** | LoE=1  N=2  **[3]** | N=3  **[3]** | N=3  **[3]** | N=2  **[2]** | LoE=1  N=3  **[4]** | **[0]** | **[0]** | **[0]** | LoE=1  **[1]** | **[0]** | **[0]** | **[0]** |

Key: LoE=Level of evidence
