# Supplementary material for: Evidence for current recommendations concerning the management of foot health for people with chronic long-term conditions: a systematic review
Source: J Foot Ankle Res. 2017 Nov 22;10:51. doi: 10.1186/s13047-017-0232-3 (PMC5700544; doi:10.1186/s13047-017-0232-3)
Supplement: Supplementary file 4 — Levels of evidence where reported from included studies. (DOCX 41 kb) [file 13047_2017_232_MOESM4_ESM.docx]

**Supplementary file 4: Podiatry evidence systematic review**

## **Appendix D:** Levels of Evidence where reported from Included Studies

| **URN** | **Author(s)** | **Themes** | **Core Podiatry**  **[A]** | **Foot ulcer care**  **[B]** | **Foot health education**  **[C]** | **Preventive care or assessments [D]** | **Preventive care advancements or assessments [E]** | **Clinical interventions**  **[F]** | **Podiatric Surgery**  **[G]** | **Other**  **[H]** | **Pain meds. [I]** | **Antibiotics**  **[J]** | **Ortho-paedic surgery**  **[K]** | **Anti-fungal meds.**  **[L]** | **Footwear**  **[M]** | **Grading System** |
| --- | --- | --- | --- | --- | --- | --- | --- | --- | --- | --- | --- | --- | --- | --- | --- | --- |
| G54 | AHRQ | B,C,D,F,J,M |  | [A-C] | [C,  Con.] | [C] |  | [A-C] |  |  |  | [B] |  |  | [Con.] | 7 |
| G56 | AHRQ | B,C,D,E |  | [Ia-IV] | [Ia] | [Ia-IV] | [IV] |  |  |  |  |  |  |  |  | 16 |
| G58 | AHRQ | B,C,D,F,J,M |  | [A-D] | [B] | [A,B,D^CPG^] |  | [B] |  |  |  | [D^CPG^] |  |  | [B] | 5 |
| G59 | AHRQ | D |  |  |  | [AI] |  |  |  |  |  |  |  |  |  | 21 |
| G63 | AHRQ | D,E |  |  |  | [C1] | [B1, CIIa] |  |  |  |  |  |  |  |  | 15 |
| G64 | AHRQ | C,F,I,K |  |  | [I] |  |  | [I] |  |  | [A,C,I] |  | [I] |  |  | 17 |
| G65 | AHRQ | C,D,M |  |  | [D] | [B] |  |  |  |  |  |  |  |  | [B] | 5 |
| G71 | AHRQ | F,I |  |  |  |  |  | [AIa] |  |  | [5D] |  |  |  |  | 22 |
| G76 | Ameen et al | L |  |  |  |  |  |  |  |  |  |  |  | [I-] |  | 5 |
| H18 | American Diabetes Association | A,B,C,D,E | [C] | [B] | [B] | [B] | [C] |  |  |  |  |  |  |  |  | 10 |
| I3 | Baker et al | A,B,C,D,E,F | [EO] | [B, C, EO] | [EO] | [C, EO] | [C] | [B,C,D] |  |  |  |  |  |  |  | 6d |
| E89 | Braun.L., Kim.P.J., Peters.E.J., Lavery.L.A., and Wound healing society. | B,D,E,K |  | [II] |  | [II] | [II] |  |  |  |  |  | [I-II] |  |  | 3 |
| I8 | Bus.S.A., Armstrong.D.G., van Deuren.R.W., Lewis.J.E., Caravaggi.P.R., and International working group on the diabetic foot | B,F,G |  | [Strong, high/  weak, mod/  weak, low] |  |  |  | [Strong, mod/  strong, low/  weak, low] | [Weak, low] |  |  |  |  |  |  | 2 |
| I9 | Bus.S.A., van Netten.J.J., Lavery.L.A.,Monteiro-Soares.M., Rasmussen.A., Jubiz.Y., Price.P.E., and International working group on the diabetic foot | A,B,C,F | [Strong, low] | [Strong, low] | [Weak, low] |  |  | [Strong, low] |  |  |  |  |  |  |  | 2 |
| H106 | Canadian Diabetes Association | B,C,D |  | [C3] | [B2] | [B2, D4, D Con.] |  |  |  |  |  |  |  |  |  | 9 |
| H76 | Canadian Diabetes Association | B,C,D,J,M |  | [D Con.] | [B2] | [D4, D Con.] |  |  |  |  |  | [D Con.] |  |  |  | 9 |
| G111 | DoHA | C |  |  | [A] |  |  |  |  |  |  |  |  |  |  | 6c |
| G123 | EULAR | D,E |  |  |  | 93 (88-98) | 86 (79-94) |  |  |  |  |  |  |  |  | 13 |
| G124 | EULAR | F |  |  |  |  |  | 91 (86-97),  95 (91-99) |  |  |  |  |  |  |  | 13 |
| E58 | Forestier.R., Andre-Vert.J., Guillez.P., Coudeyre.E., Lefevre-Colau.M.M., Combe.B., Mayoux-Benhamou,M.A. | A,C,F | [Con.] |  | [B] |  |  | [EO] |  |  |  |  |  |  |  | 18 |
| I16 | Frankel.A., Kazempour-Ardebili.S., Bedi.R., Chowdhury.T.A., et al | B,C,D,F |  | [1D] | [1D] | [1B,1C,1D] |  | [1C] |  |  |  |  |  |  |  | 11 |
| I17 | Game.F.L., Apelqvist.J., Attinger.C., Hartemann.A., Hinchliffe.R.J., Londahl.M., Price.P.E., Jeffcoate.W.J., International working group on the diabetic foot | B,F |  | [Strong, low/  strong, mod] |  |  |  | [Strong, low/  weak, mod] |  |  |  |  |  |  |  | 2 |
| E59 | Gossec et al | F |  |  |  |  |  | [C,1b/4] |  |  |  |  |  |  |  | 1 |
| G127 | Guidance: National Service Framework: Diabetes | A,C,F,M | [1] |  | [1] |  |  | [1] |  |  |  |  |  |  | [1] | 4 |
| H34 | Hinchcliffe.R.J., Brownrigg.J.R.W., Apelqvist.J., Boyko.E.J., Fitridge.R., Mills.J.L., Reekers.J., et al | D,E |  |  |  | [Strong; low] | [Strong; low] |  |  |  |  |  |  |  |  | 2 |
| I20 | Hinchliffe.R.J., Brownrigg.J.R., Apelqvist.J., Boyko.E.J.Fitidge.R., Mills.J.L., Reekers.J., Shearman.C.P., Zierler.R.E., Schaper.N.C., International working group on the diabetic foot | B,D |  | [Strong, mod-low] |  | [Strong, low] |  |  |  |  |  |  |  |  |  | 2 |
| I21 | Hingorani.A., LaMuraglia.G.N., Henke.P., Meissner.M.H., Loretz.L., Zinszer.K.M., Driver.V.R., Frykberg.R., Carman.T.L., Marston.W., Mills.J.L.Sr., Murad.M.H. | B,D,E |  | [1B,1C,2B,2C] |  | [1B, 1C] |  | [1B, 1C, 2C] |  |  |  |  |  |  |  | 2 |
| E16 | Hutchinson.A.., McIntosh.A., Feder.G.,Home.P.D.,Young.R. | B,D,F,J |  | [Ib] | [N] | [Ib] |  | [Ib] |  |  |  | [IIa-III] |  |  |  | 1 |
| I34 | Isei.T., Abe.M., Nakanishi.T., Matsuo.K., Yamasaki.O., Asana.Y., Ishii.T., et al | A,B,D,E,F,J,M | [B] | [C1] |  | [B] | [B,B] | [B,C1] |  |  |  | [B,C2] |  | [B] |  | 14 |
| H41 | Jirsch.A.T., Haskal.Z.J., Hertzer.N.R., Bakal.C.W., Creager.M.A., Halperin.J.L., Hirazka.L.F., Murphy.W.R.C., Olin.J.W., et al | A,D,E | [B1] |  |  | [C1] | [B1, CIIa] |  |  |  |  |  |  |  |  | 15 |
| I36 | Lavery.L.A., Davis.K.E., Berriman.S.J., Braun.L., Nichols.A., Kim.R.J., Margolis.D., Peters.E.J., Attinger.C | B,F,J |  | [I-III] |  |  |  | [I] |  |  |  | [II] |  |  |  | 3 |
| I37 | Lipsky.B.A., Aragon-Sanchez.J., Diggle.M., Embil.J., Kono.S., Lavery.L., et al | J |  |  |  |  |  |  |  |  |  | [Strong, high-low] |  |  |  | 2 |
| E2 | Lipsky.B.A., Berendt.A.R., Cornia.P.B., Pile.J.C., Peters.E.J.G., Armstrong.D.G., Gunner Deery.H., Embil.J.M., Joseph.W.S., Karchmer.A.W., Pinzur.M.S., and Senneville.E. | B,D,E,F,J |  | [Strong, high- low/weak, low] |  | [Strong, low/  strong, mod] | [Strong, moderate/  weak, low] | [Strong, mod/  strong, low] |  |  |  | [Strong, mod/  strong, low/  weak, low] |  |  |  | 2 |
| H85 | Massachusetts Department of Public Health | B,C,D,E,F |  | [B] | [B] | [B] | [C] | [B] |  |  |  |  |  |  |  | 10 |
| H113 | Meltzer et al | B,D,J |  | [D Con.] |  | [A1, D4, D Con.] |  |  |  |  |  | [D Con.] |  |  |  | 9 |
|  | Ministerio de Sanidad y Consum | B,C,D,F,J,M, |  | [D] | [B] | [B,D^CPG^] |  | [A] |  |  |  | [D^CPG^] |  |  | [B] | 5 |
| E50 | Moncada.L. van Voast | C,D,M |  |  | [N] | [A-B] |  |  |  |  |  |  |  |  | [B] | 19 |
| H74 | Norgren.L., Hiatt.W.R., Dormandy.J.A., Nehler.M.R., Harris.K.A., Fowkes.F.G., Rutherford.R.B., TASC II working group | D,E |  |  |  | [C] | [B] |  |  |  |  |  |  |  |  | 8 |
| H32 | Orsted.H.L., Searles.G.E., Trowell.H., Shapera.L., Miller.P., Rahman.J. | B,C,D,F,J,M |  | [Ia-IV] | [IV] | [Ia-IV] |  | [Ia-IV] |  |  |  | [N] |  |  | [Ia-IV] | 16 |
| G109 | RACGPs | D,F |  |  |  | [GPP] |  | [N] |  |  |  |  |  |  |  | 6b |
| G82 | RACGPs | A,B,C,D,E,F,M | [C] | [C] | [C] | [C] | [N] | [B] |  |  |  |  |  |  | [C] | 6d |
| H27 | Registered nurses' association | B,C,D,E,F,M |  | [Ia-IV] | [Ia-IV] | [Ia-IV] | [IV] | [Ia-IV] |  |  |  |  |  |  | [Ia-IV] | 16 |
| H28 | Registered nurses' association | B,C,D,E,F,M |  | [IV] | [Ib,IV] | [Ib,IV] | [IV] | [IV] |  |  |  |  |  |  | [IV] | 16 |
| G100 | SIGN | D |  |  |  | [GPP] |  |  |  |  |  |  |  |  |  | 5 |
| G122 | SIGN | D,E |  |  |  | [N] | [GPP] |  |  |  |  |  |  |  |  | 5 |
| G77 | SIGN | F |  |  |  |  |  | [C] |  |  |  |  |  |  |  | 5 |
| G98 | SIGN | A,C,D,E | [GPP] |  | [N] | [GPP] | [2-] |  |  |  |  |  |  |  |  | 5 |
| H111 | SIGN 116 | C,D,E,F,J |  |  | [B] | [B] | [GPP] | [B] |  |  |  | [GPP] |  |  |  | 5 |
| H112 | SIGN 55 | A,C,D,F,J | [C] |  | [B] | [D] |  | [B] |  |  |  | [GPP] |  |  |  | 5 |
| E42 | Steed.D.L., Attinger.C., Brem.H., Colaizzi.T., Crossland.M., et al | A,B,C,D,E,F | [3, 3] | [2] | [2] | [1] | [1] | [2] |  |  |  |  |  |  |  | 3 |
| H48 | Steed.D.L., Attinger.C.,. Colaizzi.T., Crossland.M., Franz.M., Harkless.L., Johnson.A., et al | B,F,G,M |  | [I-III] |  |  |  | [I] | [II] |  |  | [II] |  |  |  | 3 |
| E31 | Thomas.J.L., Christensen.J.C., Kravitx.S.R., Mendicino.R.W., Schuberth.J.M., Vanore.J.V., et al | D,F,G,I |  |  |  | [N] |  | [B, B, I] | [B,I] |  | [B] |  |  |  |  | 12 |
| H66 | VA/DoD | A,B,C,D,M | [I] | [I] | [B] | [B,I] |  |  |  |  |  |  |  |  | [I] | 20 |
| 151 | Weinstein, et al | F |  |  |  |  |  | [AI] |  |  |  |  |  |  |  | 15 |
| H57 | Whitney et al | B,F,G,J |  | [I-III] |  |  |  | [I-II] | [I-III] |  |  | [I-II] |  |  |  | 3 |
| E22 | Wraight et al | B,D,F,J |  | [III, III-1, EO, EO+1] |  | [I-IV] |  | [EO] |  |  |  | [III-IV] |  |  |  | 6 |

Key: Con=Consensus; EO=Expert opinion; GPP=Good practice point; N=Narrative
