# Supplementary material for: Evidence for current recommendations concerning the management of foot health for people with chronic long-term conditions: a systematic review
Source: J Foot Ankle Res. 2017 Nov 22;10:51. doi: 10.1186/s13047-017-0232-3 (PMC5700544; doi:10.1186/s13047-017-0232-3)
Supplement: Supplementary file 5 — Levels of Evidence for Recommendations concerning Foot Health Management. (DOCX 28 kb) [file 13047_2017_232_MOESM5_ESM.docx]

**Supplementary file 5: Podiatry evidence systematic review**

## **Appendix E:** Levels of Evidence for Recommendations concerning Foot Health Management

| **AGREE** | **[A]** | **[B]** | **[C]** | **[D]** | **[E]** | **[F]** | **[G]** | **[H]** | **[I]** | **[J]** | **[K]** | **[L]** | **[M]** |
| --- | --- | --- | --- | --- | --- | --- | --- | --- | --- | --- | --- | --- | --- |
| **1** |  |  |  |  |  | [C, Ib-IV^1^] |  |  |  |  |  |  |  |
| **2** |  |  |  |  |  |  |  |  |  |  |  |  |  |
| **3** | Strong,low^2^ | Strong, mod-low(2)^2^  Strong,low^2^  Strong, high/ weak, mod/weak, low^2^ | Weak,low^2^ | Strong,low^2^ |  | Strong,low & weak,mod^2^  Strong,mod/ strong,low/ weak,low^2^  Strong,low^2^ | Weak,low^2^ |  |  |  |  |  |  |
| **4** | Level 3,3^3^  Level 1^4^  Grade GPP^5^ | Grade Ib^1^  Level III, III-1, EO, EO+1^6a^  Level 2^3^  Level A-C^7^  Grade A-D^5^  Level I-III^3^  Grade/Level D,Con.^9^  Level B^10^  Grade 1D^11^ | Level 2^3^  Level C Con^7^  Grade B^5^  Grade/Level B2^9^  Level B^10^  Grade 1D^11^  Level 1^4^  Grade D^5^ | Level Ib^1^  Level I-IV^6a^  Level 1^3^  GPP (2)^5,6b^  SoR 93 (88-98)^13^  Level C^7^  Grade A,B,D^CPG 5^  Grade B^5^  Grade/Level D4 & D Cons.^9^  Level B^10^  Grade 1B,1C,1D^11^  Grade C^8^ | Level 1^3^  Grade GPP^5^  SoR 86 (79-94)^13^  Level 2-^5^  Grade B^8^  Grade C^10^ | Grade Ib^1^  Grade EO^6a^  Grade B,B,I^12^  Level 2^3^  SoR 91(86-97) & 95(91-99)^13^  Level 1^4^  Grade A-C^7^  Grade B^5^  Level I^3^  Grade B^10^  Grade 1C^11^ | Level II^3^  Grade B,I^12^ |  | Grade B^12^ | Level IIa-III^1^  Level III-IV^6a^  Grade B^7^  GradeD^CPG 5^  Level II^3^  Grade/Level D,Con.^9^ |  |  | Grade B^5^  Grade B^5^  Level 1^4^ |
| **5** | Level B^14^ | Level C1^14^  Grade 1B,1C,2B,2C^2^  Level I-III^3^  Grade/Level D Con.^9^  Level II^3^ |  | Level B^14^  Grade 1B,1C^2^  Grade/Level A1, D4, D Con.^9^  Level II^3^ | Level B,B^14^  Level II^3^ | Level B,C1^14^  Grade 1B,1C,2C^2^  Level I-II^3^ | Level I-III^3^ |  |  | Level C2,B^14^  Level I-II^3^  Grade/Level D Con.^9^  Strong, high-low^2^ | Level I-II^3^ | Level B^14^ |  |
| **6** | Grade C^6d^  Level C^10^  Grade I^20^  Grade B1^15^ | Grade C^6d^  Level B^10^  Grade I^20^  Strong, high-low & weak, low^2^  Level Ia-IV^16^  Level IV^16^ | Grade C^6d^  Level B^10^  Grade B^20^  Level Ia-IV^16^  Level Ib,IV^16^  Level I^17^  Grade B^18^ | Grade C^6d^  Level B^10^  Grade B,I^20^  Grade C1^14^  Level Ia-IV^16^  Level Ib, IV^16^  Strong,low & strong, mod^2^  Grade A-B^19^ | Level C^10^  Grade B1, CIIa^14^ Level C^15^  Level IV^16^  Level IV^16^  Strong,mod & weak,low^2^ | Grade B^6d^  Grade EO^18^  Strong,mod & strong,low^2^  Grade A1^15^  Level I^17^  Level Ia-IV^16^  Level IV^16^ |  |  | Level A,C,I^17^ | Strong,mod & strong,low & weak,low^2^ | Level I^17^ | Level 1-^5^ | Grade C^6d^  Grade I^20^  Level Ia-IV^16^  Level IV^16^  Grade B^19^ |
| **7** | Grade C^5^  Grade EO^6d^ | Grade B,C,EO^6d^  Level Ia-IV^16^  Grade/Level C3^9^  Level Ia-IV^16^  Grade D^5^ | Grade B^5^  Grade EO^6d^  Level Ia^16^  Grade/Level B2^9^  Level IV^16^  Grade B^5^  Grade A^6c^  Grade B^5^ | Grade D^5^  Grade C,EO^6d^  Level Ia-IV^16^  Grade/Level B2,D4, Cons.^9^  Level Ia-IV^16^  Grade B,D^CPG 5^  Grade GPP^5^  SR/Level AI^21^  Grade C1^15^  Grade B^5^  Strong,low^2^ | Grade C^6d^  Level IV^16^  Grade B1 & CIIa^15^  Strong,low^2^  Grade=GPP^5^ | Grade B^5^  Grade B,C,D^6d^  Level Ia-IV^16^  Grade A^5^  Level/Grade AIa^22^  Grade C^5^  Grade B ^5^ |  |  | Level/Grade 5D^22^ | Grade GPP^5^  Grade D^CPG 5^  Grade GPP^5^ |  |  | Level Ia-IV^16^  Grade B^5^ |
| **/** |  | Level I-III^3^ |  |  |  | Level I^3^ |  |  |  | Level II^3^ |  |  |  |

**Key to Grading Systems used by Included Studies:**

1=(Shekelle, P.G et al, 1999)

2=Grading of recommendations assessment, development and evaluation (GRADE)

3=(Steed, D.L et al,2008)

4=(National servive framework for diabetes, 2001)

5=Scottish Intercollegiate guidelines network (SIGN)

6=NMHRC (6a=NHMRC (1999); 6b= NMHRC (2005-2007); 6c= NMHRC (2007); 6d= NMHRC (2009))

7=(Agency for healthcare research and quality (AHRQ), 2012)

8=(Norgren, L et al, 2007)

9=Canadian diabetes association

10=American diabetes association

11=(Frankel, A et al, 2016)

12=(Thomas, J.L et al, 2010)

13=European league against Rheumatism (EULAR)

14=(Isei, I et al, 2016)

15=American Heart Association (AHA)/American Stroke Association (ASA)

16=Registered nurses’ association (RNAO)

17=(Agency for healthcare quality and research (AHRQ), 2011 – Adapted from Cochrane collaboration back group)

18= HauteAutorité de santé, 2000

19=Strength of recommendation taxonomy (SORT)

20= (Va/Dod clinical practice guideline, 2010 - Based on U.S. preventative services task force (USPSTF))

21=(Agency for healthcare research and quality (AHRQ), 2012)

22=Oxford centre for evidence-based medicine (after the 2009 modification)
